# From emergency adaptation to institutional resilience: a policy and practice review of Somalia's health system, 1991–2026

**DOI:** 10.3389/frhs.2026.1934074

**Published:** 2026-09-01

**Authors:** Nor Haji Osman, Hamdi Mohamud Osman, Abdifatah Nour Rage, Abdiweli Mohamed Abdi, Mohamed Ismail Iman, Aweys Ahmed Moallim, Abdirahman Mohamed Jimale, Mohamed Ahmed Ali, Ismail Dahir Ahmed, Abdikarim Abdi Adam, Abdirahman Ahmed Mohamud, Siham Mohamed Dhakane, Osman Mohamed Mohamud

**Affiliations:** 1 Department of Public Health, Faculty of Health Sciences, Al Hayat Medical University, Mogadishu, Somalia; 2 Department of Policy and Planning, Directorate of Health and Human Services, Mogadishu, Somalia; 3 Noncommunicable Diseases Section, Ministry of Health and Human Services, Mogadishu, Somalia; 4 Department of Social Sciences, Faculty of Humanities and Social Sciences, Salaam University, Mogadishu, Somalia; 5 Department of Medicine, Faculty of Medicine and Surgery, Al Hayat Medical University, Mogadishu, Somalia; 6 Department of Administration, Human Resources and Capacity Building, Directorate of Health and Human Services, Mogadishu, Somalia; 7 Department of Environmental Health and Climate, IPC Section, Federal Ministry of Health and Human Services, Mogadishu, Somalia; 8 Department of Health, Directorate of Health and Human Services, Mogadishu, Somalia; 9 Department of Public Health, Faculty of Health Sciences, Zamzam University of Science and Technology, Mogadishu, Somalia; 10 Department of Medicine, Faculty of Medicine and Health Sciences, Zamzam University of Science and Technology, Mogadishu, Somalia

**Keywords:** climate-health preparedness, community health workers, fragile and conflict-affected settings, health financing, health policy, health-system resilience, primary health care, Somalia

## Abstract

Health-system resilience is a major policy concern in fragile and conflict-affected settings, where recurrent shocks intersect with chronic weaknesses in governance, financing, workforce, service delivery, information systems, and supply chains. This policy and practice review synthesizes 51 peer-reviewed and institutional sources published or issued between 1990 and 3 July 2026 to examine how Somalia's health system has responded to three decades of conflict, climatic emergencies, displacement, outbreaks, and humanitarian intervention. Sources were identified through targeted searches of PubMed/MEDLINE, Google Scholar, and authoritative institutional repositories and interpreted using the World Health Organization health-system building blocks and contemporary resilience concepts. The review distinguishes absorptive, adaptive, and transformative resilience from temporary emergency adaptation and donor-dependent response capacity using explicit decision rules concerning service continuity, duration, financing and ownership, integration into routine systems, and evidence of learning and accountability. Somalia has accumulated important resilience-enabling capacities, including community-based surveillance, outbreak-response learning, expansion of the District Health Information Software 2, national strategic planning, and the Community Health Strategy 2025–2029. However, chronic underfinancing, fragmented governance and service delivery, workforce shortages, weak supply chains, incomplete private-sector stewardship, inequitable access, and uneven primary health-care readiness continue to limit institutional resilience. The review proposes seven actionable priorities: a unified national resilience framework; predictable and aligned financing; primary health-care readiness and continuity planning; professionalized community health platforms; private-sector integration into regulation, referral, and reporting; climate-health, nutrition, and water, sanitation and hygiene preparedness; and an operational resilience dashboard linked to decision triggers and actor accountability. These priorities are policy inferences derived from Somalia-specific operational evidence, national strategies, and wider fragile-setting literature rather than causally proven interventions. Given binding fiscal and data constraints, implementation should be phased, costed, and begin with functions and indicators that can be sustained through existing routine systems. Somalia should therefore not yet be described as having a fully resilient health system. Durable resilience will depend on embedding recurrent emergency adaptations within financed, governed, measurable, equitable, and routinely functioning primary health-care and public-health institutions.

## 1 Introduction

Health-system resilience has become a central concern for countries facing epidemics, conflict, climate shocks, economic stress, and displacement. WHO frames resilient health systems as systems able to prevent, prepare for, detect, adapt to, respond to, and recover from public health threats while maintaining essential health services (1). OECD similarly emphasizes the capacity to foresee, absorb, recover from, and adapt to shocks such as pandemics, climate change, geopolitical conflict, and other systemic threats (2). Peer-reviewed resilience literature further emphasizes that health-system resilience depends on governance, learning, flexibility, adaptation, equity, quality of care, and the capacity to absorb, adapt and transform while maintaining essential services (3–5). Importantly, resilience should not be treated as a way to shift the burden of survival onto communities or frontline health workers; recent critiques warn that resilience strategies can reproduce inequity if they ignore power relations, accountability, and who bears the real costs of adaptation (6). These definitions are especially relevant to fragile and conflict-affected settings, where shocks rarely occur as isolated events but instead interact with chronic weaknesses in governance, financing, workforce availability, supply chains, information systems and service delivery (7).

Somalia is one of the clearest examples of a health system forced to operate under prolonged stress. Since the collapse of the central state in 1991, the country has experienced conflict, weak public institutions, repeated drought and flood emergencies, mass internal displacement, food insecurity, communicable disease outbreaks, and a large mixed public–private and humanitarian service environment (8–11). The health sector has relied heavily on non-governmental organizations, United Nations agencies, donors, diaspora support, private providers, and local community structures to maintain essential services (12). This has created adaptive capacity, but also fragmentation and uneven standards.

The Somalia Health Sector Strategic Plan III 2022–2026 seeks to improve access to affordable, equitable, and quality health services, is built around the revised Essential Package of Health Services, and uses WHO's six health-system building blocks as an organizing framework (10, 13). The 2025–2029 Community Health Strategy, launched nationally in 2026, further signals a shift toward a government-led and professionalized community health platform centered on female health workers who link families with primary health-care services (14). These reforms are important because health-system resilience cannot be reduced to short-term emergency response. Resilience requires sustained primary health care functions that remain operational before, during and after shocks, including integrated service delivery, routine health information and surveillance, reliable financing, essential medicines and supplies, health workforce capacity, effective governance and community trust (15–18). Recent PHC resilience scholarship similarly shows that resilient primary care depends on coordinated governance, financing, workforce preparedness, medicines and infrastructure readiness, information systems, community engagement, adaptive service delivery and continuous learning, rather than isolated emergency projects (19, 20).

Despite these reforms, Somalia's health-system resilience remains insufficiently synthesized in both peer-reviewed and policy literature. Existing evidence tends to document specific shocks, outbreaks, humanitarian programmes, financing constraints, community-health interventions, or sector plans, rather than connecting three decades of crisis response with the institutional capacities required for long-term resilience (21–23). This review therefore distinguishes four related but analytically different phenomena. Temporary emergency adaptation refers to time-limited coping or service-continuity measures activated during a shock. Donor- or partner-dependent response capacity refers to activities whose continuation depends substantially on externally financed projects, logistics, personnel, commodities, or parallel reporting arrangements. Institutional resilience refers to capacities embedded in government-led rules, recurrent financing, routine workforce and supply systems, information architecture, accountability, and learning mechanisms that remain functional between shocks. Institutional transformation refers to formal and sustained changes in governance, financing, organizational structures, or service-delivery arrangements that address weaknesses exposed by crises. Evidence could contribute to more than one category, but it was interpreted according to the dominant characteristics and degree of institutionalization described by the source. This distinction is important because short-term coping or externally supported emergency response should not automatically be interpreted as durable institutional resilience (21, 22, 24). The central policy question is therefore how repeated emergency adaptation can be converted into institutional resilience through stewardship, financing, PHC readiness, community systems, private-sector governance, climate-health integration, and routine measurement.

Accordingly, this review aims to (1): synthesize evidence on how Somalia's health system has responded to major shocks since 1991; (2) distinguish temporary adaptation and partner-dependent response from institutional resilience; and (3) identify measurable, actor-specific priorities for converting recurrent emergency response into durable system capacity.

## 2 Review approach and analytical framework

### 2.1 Review approach

This article is a narrative policy review. It synthesizes peer-reviewed literature, national strategic documents, institutional reports, emergency response updates, and health-system assessments relevant to Somalia from 1990 to 3 July 2026. The review was designed to support policy interpretation and system-level learning rather than estimate pooled effects. It was not conducted as a systematic review; therefore, no meta-analysis, formal risk-of-bias scoring, or PRISMA flow diagram was produced. A PRISMA diagram would have implied an exhaustive database search, duplicate screening, and complete database-level flow counts that were not part of this purposive design. Instead, reporting transparency was strengthened in line with the Scale for the Assessment of Narrative Review Articles (SANRA) by specifying the review rationale, search sources and dates, eligibility categories, extraction fields, analytical decision rules, evidentiary limitations, and the basis for policy interpretation (25). The final synthesis included 51 sources retained in the reference list. Database-level hit counts were not treated as evidence of inclusion; source selection focused on relevance, credibility, policy importance, and triangulation across peer-reviewed and institutional evidence.

Searches were conducted and updated between 1 June and 3 July 2026. Sources were identified through targeted searches of PubMed/MEDLINE, Google Scholar, WHO, UNICEF, World Bank, Global Financing Facility, United Nations Office for the Coordination of Humanitarian Affairs, Somalia Ministry of Health and Human Services documents, WHO Regional Office for the Eastern Mediterranean materials, and selected grey-literature repositories. Sample search strategies included: PubMed/MEDLINE: Somalia AND (health system OR resilience OR primary health care OR health financing OR health workforce OR DHIS2 OR surveillance OR cholera OR drought OR famine); Google Scholar: Somalia health system resilience conflict humanitarian emergencies primary health care; and institutional searches combining Somalia with HSSP, EPHS, Community Health Strategy, cholera response, drought mortality, health financing, and health information system. Reference lists of highly relevant articles and institutional reports were also screened to identify additional sources.

Eligibility was organized into four overarching source categories. First, Somalia-specific empirical and health-system evidence was eligible when it addressed governance, financing, workforce, primary health care, service delivery, surveillance, health information, supply chains, WASH, nutrition, climate-health vulnerability, equity, or emergency response. Second, national policy and institutional documents were eligible when they defined strategic direction, standards, financing arrangements, implementation responsibilities, or nationally relevant indicators. Third, humanitarian and emergency reports were eligible when they documented major shocks, operational responses, service disruption, mortality, morbidity, displacement, or funding constraints using identifiable methods or institutional reporting systems. Fourth, conceptual, comparative, and methodological literature was eligible when it informed interpretation of resilience, fragile and conflict-affected settings, WHO health-system building blocks, or narrative and framework synthesis. Priority was given, in order, to primary Somalia-specific peer-reviewed studies and official national documents, authoritative institutional reports with transparent methods, and wider conceptual evidence used to interpret gaps for which Somalia-specific studies were limited. Older sources were retained when they documented historically important shocks such as state collapse, famine, polio, cholera, or drought-related mortality.

Sources were excluded when they were not relevant to Somalia or health-system resilience, focused only on clinical management without health-system implications, duplicated evidence available from a primary or more authoritative source, lacked sufficient methodological or institutional credibility, or presented opinion without a clear connection to the review question. The first author conducted the initial source identification, eligibility screening, and extraction. Co-authors checked the final source set, key quantitative claims, and sources for which eligibility, interpretation, or policy relevance was uncertain. Uncertain decisions were recorded with the reason for concern and discussed among the authors until consensus was reached. The review did not use independent duplicate screening or calculate inter-rater agreement, and this is acknowledged as a limitation. When disagreement persisted, preference was given to primary official records or peer-reviewed sources; otherwise, the claim was qualified as uncertain, identified as single-source evidence, or excluded. Institutional claims were triangulated with peer-reviewed studies, official records, or multiple independent sources where feasible rather than treated as automatically equivalent evidence.

Data were extracted into a structured thematic matrix covering source type, year, setting, shock or health-system function, key findings, quantitative estimate where applicable, resilience capacity described, vulnerability identified, ownership and financing arrangement, degree of integration into routine systems, and policy implication. This approach is consistent with thematic and framework-based synthesis methods, which are suitable for organizing heterogeneous peer-reviewed and policy evidence while preserving contextual interpretation (26–28). To improve reproducibility of the central conceptual distinction, each relevant response was interpreted across five decision domains: (1) whether essential services or public-health functions continued during the shock; (2) whether the response persisted beyond the immediate emergency; (3) whether financing, leadership, logistics, and accountability were primarily domestic, jointly governed, or externally dependent; (4) whether the response was integrated into routine governance, workforce, supply-chain, and information systems; and (5) whether it generated sustained learning, equity protection, or institutional accountability. A response was classified as temporary emergency adaptation when continuity depended mainly on time-limited coping measures; as donor-dependent response capacity when external project inputs were indispensable to continued operation; as absorptive or adaptive capacity when essential functions were maintained or modified during a shock; and as transformative or institutional resilience only when changes were embedded in routine rules, budgets, organizations, and systems between shocks. These classifications were interpretive categories rather than validated numerical scores. WHO's health-system building blocks were then used to organize findings across leadership and governance, health financing, health workforce, service delivery, health information systems, and medical products, vaccines, and technologies (29), while resilience concepts guided the absorptive, adaptive, and transformative interpretation (30). Quantitative figures from institutional reports were treated cautiously because Somalia's information system remains fragmented, DHIS2 reporting is still largely public-sector focused, private-sector reporting is not fully institutionalized, routine data quality is uneven, and birth, death, and mortality systems require strengthening (31, 32). To improve numerical precision, source-reported point estimates are reproduced as reported and identified as estimates where applicable; ranges, upper or lower bounds, and scenario-specific values are retained when they convey uncertainty in the original source.

Generative artificial intelligence was used only after the scientific content had been drafted, for language editing, structural refinement, document formatting, and reference-style consistency. The authors independently checked the factual content, citations, and references and retained full responsibility for the manuscript.

### 2.2 Conceptual framing

A resilient health system is not a system that avoids shocks. It is a system that can maintain essential services, protect vulnerable populations, learn from disruptions, and transform weaknesses exposed by crises. For Somalia, the operational level concerns observable continuity during outbreaks, drought, conflict, and displacement, including whether services, surveillance, referrals, commodities, and workforce functions remain available. The institutional level concerns whether the response is governed through accountable national or subnational arrangements, supported by recurrent and predictable financing, integrated into routine workforce, supply-chain, and information systems, and sustained between emergencies. Operational success was therefore not treated as institutional resilience unless the evidence also demonstrated persistence, ownership, integration, and learning.

This distinction matters because Somalia has repeatedly shown operational adaptation without fully institutionalizing those adaptations. For example, reported cholera case-fatality ratios fell across the 2017–2019 period, and community health workers were central to COVID-19 surveillance and household screening. These observations indicate operational learning and reach, but they do not by themselves establish durable resilience or prove that any single intervention caused the observed outcome. When response systems depend mainly on short-term donor funding, external logistics, temporary campaigns, or parallel information systems, the dominant classification remains project-dependent adaptation rather than institutional resilience.

**Figure 1:**
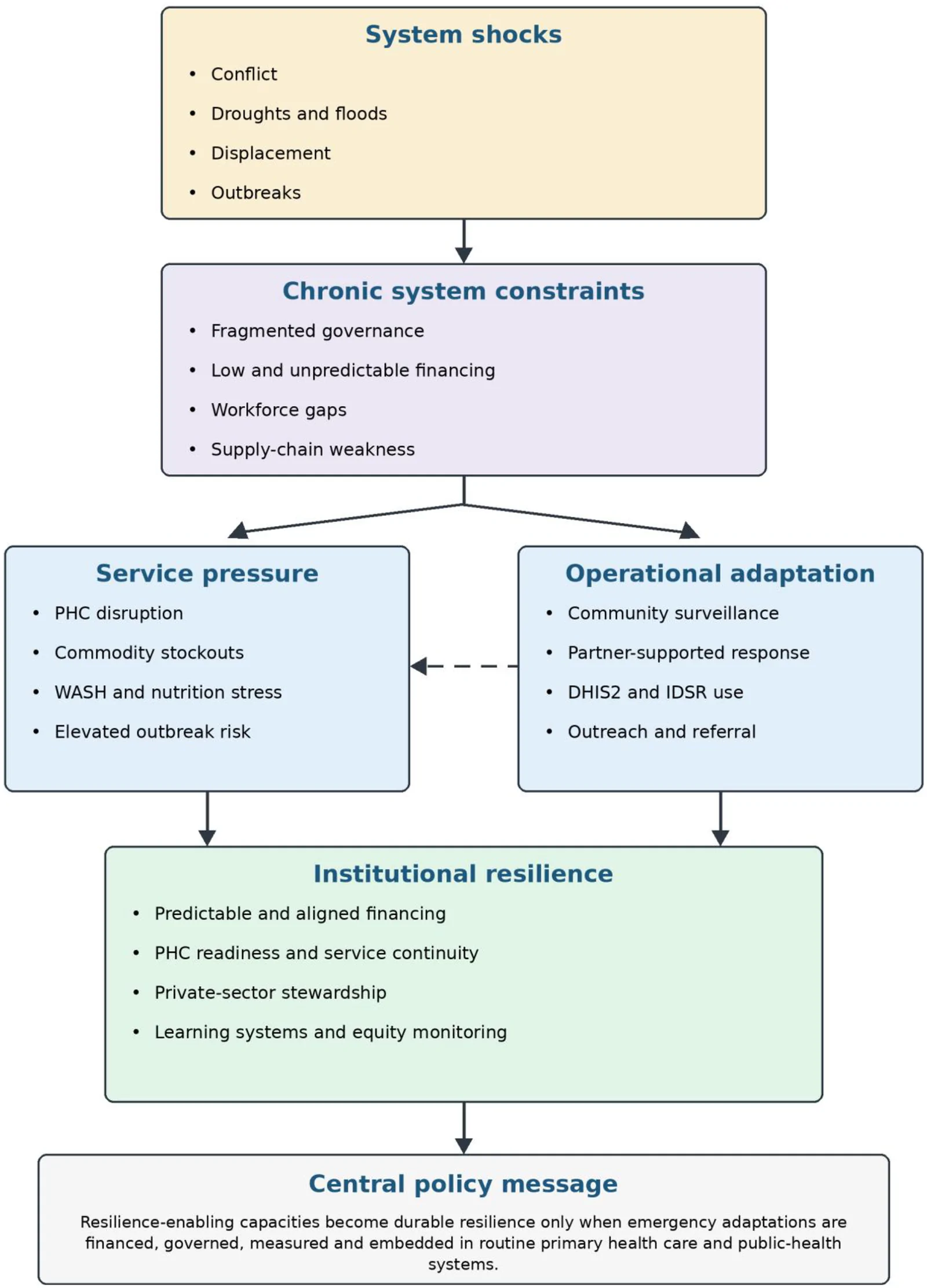
Pathway from recurrent shocks to institutional health-system resilience in Somalia.

Figure 1 presents the proposed pathway from recurrent shocks and chronic system constraints to operational adaptation and, when these adaptations are financed, governed, measured, and embedded in routine systems, to institutional resilience.

## 3 Assessment of resilience capacities and policy implications

### 3.1 Three decades of shocks and responses

Somalia's post-1991 health trajectory reflects a repeated cycle of institutional collapse, emergency substitution, partial reconstruction, and recurrent shock. The collapse of the central state in 1991 severely weakened public-sector infrastructure, regulation, financing, workforce management, and national stewardship of health service delivery (33, 34). In the years that followed, non-governmental organizations, United Nations agencies, private clinics, diaspora-supported providers, and local community actors became essential providers of health services, often filling gaps left by the weakened public sector (33). While this pluralistic service landscape helped preserve access to basic care during prolonged crisis, it also contributed to fragmentation, donor dependence, uneven quality, weak regulation, and limited government control over financing and service standards (35, 36). This emergency substitution saved lives but also embedded fragmentation as a normal operating model.

The 2011 famine remains a defining example of system failure under combined food insecurity, conflict, displacement, and delayed response. A major mortality estimation study estimated 258,000 excess deaths in southern and central Somalia during the 2010–2012 famine crisis, including 133,000 deaths among children under five (37). This famine mortality burden has also been recognized in peer-reviewed analysis of drought, armed conflict and population mortality in Somalia (38). Subsequent analyses emphasize that the famine was not only a drought event; it reflected the interaction of insecurity, market disruption, restricted humanitarian access, weak health services, and poor nutrition and WASH conditions.

Recurrent cholera and acute watery diarrhoea outbreaks demonstrate both vulnerability and operational learning in Somalia's health system. In 2017, Somalia reported 78,701 cholera cases and 1,163 deaths, with a case-fatality ratio of 1.48%; by 2019, reported cases had declined to 3,089 and deaths to four, with a case-fatality ratio of 0.13% (39). The lower reported case-fatality ratio coincided with strengthened coordination, surveillance and alert systems, epidemiological information use, risk assessment, case management, laboratory and surveillance capacity, WASH preparedness, community engagement, risk communication, and preventive oral cholera vaccination (39). However, the observational evidence cannot isolate the causal contribution of these components, and changes in outbreak magnitude, case ascertainment, access to treatment, reporting completeness, and the geographic distribution of cases may also have influenced the reported ratio. The decline is therefore interpreted as evidence consistent with improved response capacity, not as proof that the listed interventions independently caused the change. Cholera continued to re-emerge, indicating that response capacity had improved without fully resolving the environmental, displacement-related, WASH, and service-delivery vulnerabilities that sustain transmission. By the end of July 2024, UNICEF reported 16,927 cholera cases and 136 deaths across all seven states and 30 of Somalia's 74 districts, with flooding, damaged health and WASH infrastructure, malnutrition, and continued transmission since 2017 contributing to ongoing risk (40).

Community-based surveillance has also contributed to Somalia's health-system resilience. During and after the 2013 polio outbreak response, the Village Polio Volunteers programme strengthened acute flaccid paralysis surveillance and extended reporting into communities. After the programme was introduced, acute flaccid paralysis case reporting improved, and Village Polio Volunteers accounted for 40% of reported cases in 2015, demonstrating the value of community-based surveillance in settings with limited health infrastructure (41). During COVID-19, community health workers also played a major surveillance role: between March 2020 and March 2021, they identified 32.7% of suspected COVID-19 cases and substantially more contacts than facility-based surveillance, supporting case detection when facility access was constrained (42). These examples show that community platforms can extend reach during crises, but they require formal supervision, financing, training, reporting systems, and strong links to primary health care if they are to remain effective beyond emergencies.

Climate-sensitive shocks have continued. The 2021–2024 drought and flood period generated large humanitarian need, malnutrition, displacement, and disease risk. A 2025 mortality report released by Somalia's Federal Ministry of Health, WHO and UNICEF estimated that 71,100 excess deaths may have occurred in Somalia between January 2022 and June 2024 because of drought, with 41% of deaths occurring among children younger than five (43). Earlier peer-reviewed statistical work also estimated substantial excess mortality during the 2017–2018 drought period, including 44,700 excess deaths under the most likely counterfactual scenario and up to 163,800 deaths under a pessimistic scenario (38). Together, these findings show that climate shocks in Somalia rapidly translate into health-system stress, especially where food insecurity, displacement, malnutrition, weak WASH systems, and limited-service access overlap.

The mortality estimates for 2010–2012, 2017–2018, and 2022–2024 are not directly comparable and should not be read as a time trend. The 2010–2012 estimate focused on famine-affected populations in southern and central Somalia and used a specific crisis period and counterfactual framework (37). The 2017–2018 analysis covered a different period and population and reported a range generated by alternative counterfactual scenarios (38). The 2022–2024 estimate used different geographic coverage, exposure definitions, baseline assumptions, and data availability (43). Differences in surveillance, displacement, population denominators, humanitarian access, and modelling methods may account for part of the variation. In this review, the estimates are used only to demonstrate that successive crises were associated with substantial excess mortality; they are not used to infer that mortality increased or decreased between the three periods.

Climate-health resilience should therefore be treated as a cross-cutting health-system function rather than a separate emergency topic. In Somalia, droughts, floods, displacement, malnutrition, unsafe water, and cholera interact through the same vulnerable households and districts. Preparedness should link PHC readiness, nutrition surveillance, WASH response, IDSR/EWARN alerts, pre-positioned cholera and nutrition commodities, referral transport, and district contingency planning. This framing avoids treating climate shocks as isolated events and instead positions climate-health preparedness as part of routine health-system resilience.

Table 1 summarizes the major shocks, response patterns, and resilience lessons identified across the period 1991–2026.

**Table 1:** Major shocks, response patterns, and resilience lessons in Somalia, 1991–2026.

Period or shock	Health-system impact	Response or adaptive capacity	Resilience lesson
1991 state collapse	Public-sector infrastructure, regulation, workforce management, and financing weakened severely.	NGOs, private clinics, UN agencies, diaspora support, and local actors filled major service gaps.	Emergency substitution preserved access but normalized fragmentation.
2010–2012 famine	Large excess mortality, malnutrition, displacement, and outbreak risk; an estimated 258,000 excess deaths occurred in affected areas.	Food, nutrition, WASH, and emergency health response scaled up, but access constraints delayed impact.	Early warning must translate into funded, accessible, multisector action before mortality accelerates.
2013–2014 polio outbreak	Risk of renewed poliovirus transmission and surveillance gaps.	Village Polio Volunteers strengthened community-based acute flaccid paralysis reporting.	Local community surveillance can extend reach in insecure and underserved areas.
2017–2019 cholera	Large 2017 outbreak with high mortality, followed by lower reported case fatality in later waves.	Case management, WASH, surveillance, and vaccination activities were coordinated by government and partners.	Response capacity improved, but recurrent cholera shows unresolved WASH and PHC vulnerabilities.
2020–2021 COVID-19	Risk of severe disruption in a health system with limited intensive care, laboratory, and oxygen capacity.	CHWs supported surveillance, household screening, and contact identification.	Investing in community systems and basic public-health infrastructure has value across emergencies.
2022–2024 drought, floods, and cholera	Malnutrition, displacement, excess mortality, and cholera re-emergence intensified system pressure.	Humanitarian response, cholera planning, and surveillance were scaled up.	Climate-health preparedness must be linked to WASH, nutrition, PHC, and displacement planning.
2025–2029 Community Health Strategy	Opportunity to move from fragmented community projects toward a national government-led platform.	Strategy launched in 2026 with emphasis on female health workers linked to primary health care.	Transformation requires financing, supervision, commodities, and integration into routine systems.

CHW, community health worker; PHC, primary health care; UN, United Nations; WASH, water, sanitation and hygiene.

### 3.2 Governance and coordination

Governance is the core resilience constraint in Somalia. The federal system requires coordination between the Federal Government, Federal Member States, regional authorities, districts, humanitarian actors, private providers, and community structures. The HSSP III provides a strategic framework and explicitly uses the WHO building blocks, but implementation remains challenged by decentralization, variable state capacity, fragmented partner activity, and limited public stewardship over private and humanitarian service delivery. Evidence from fragile and conflict-affected settings shows that resilience depends on coordinated stewardship, accountability, community involvement, and integrated service-delivery arrangements rather than parallel projects (44).

A repeated lesson from Somalia is that parallel service delivery can be lifesaving in emergencies but problematic for resilience. When partners operate with separate funding cycles, reporting tools, salary structures, geographic priorities, and commodity systems, the country may gain short-term coverage without building durable national capacity. The Global Financing Facility investment case recognizes this problem by emphasizing alignment around the Essential Package of Health Services, financing coordination, accountability, and reduction of fragmentation (12).

### 3.3 Health financing

Financing remains a direct threat to resilience in Somalia. The Health Sector Strategic Plan III and the Somalia Health Sector Investment Case identify low, fragmented, and externally dependent financing as a central barrier to universal health coverage and health-system strengthening (10, 12). Recent secondary reporting has also raised concerns about low federal prioritization of health spending (45). However, differences in budget definitions, donor-funded projects, off-budget humanitarian expenditure, transfers, and sector classifications limit direct comparison of budget shares across years. The official 2025 Appropriation Act allocated US$91.85 million to the Ministry of Health, including US$68.42 million recorded under special-project appropriations (46). These figures describe approved federal appropriations, not actual releases, execution, total national health expenditure, or all off-budget assistance, and they therefore cannot by themselves establish the precise level of aid dependence. Household financial protection is also weak in Somalia's mixed public–private health-care market. Recent Somalia-specific evidence estimates that out-of-pocket payments account for 44.9% of total health expenditure and that only 3.5% of Somalis have health insurance coverage (47).

Recent programme-level evidence further illustrates the vulnerability created by funding shortfalls, while requiring careful interpretation of denominators. UNICEF reported that its Somalia Humanitarian Action for Children appeal was 15% funded in the third quarter of 2025 and described an 87% funding gap for the health component, alongside concern that up to one third of supported health centres could close in 2026 (48). Médecins Sans Frontières separately reported that the suspension of USAID funding was followed by closure of at least 37 health and nutrition sites in rural and urban areas around Baidoa, with increased pressure on remaining services (49). These figures refer to specific agency portfolios and affected programme areas rather than the entire national health system, but they provide concrete evidence that externally financed service platforms can contract rapidly when funding is interrupted.

The financing problem is not only the amount of money available; it is also the predictability, flexibility, execution, pooling, and alignment of funds. Short emergency grants can support acute response but are poorly suited to recurrent salaries, supervision, maintenance, medicine procurement, private-sector reporting, referral systems, and district-level preparedness. Evidence from fragile and conflict-affected settings shows that such countries commonly face lower government health expenditure, higher out-of-pocket payments, greater external dependency, and health-related impoverishment, while aid coordination often remains dominated by external actors (50, 51). Resilience therefore requires multi-year, nationally aligned financing that protects essential services during shocks, funds preparedness before emergencies occur, and strengthens domestic stewardship rather than creating or reinforcing parallel financing and delivery systems (12, 23, 50, 51).

### 3.4 Health workforce and community health systems

Somalia's health workforce remains insufficient and unevenly distributed (10, 34). Previous WHO reporting noted four doctors, nurses, and midwives per 10,000 population, far below common international density thresholds for essential service coverage (10). Workforce shortages are intensified by insecurity, migration, concentration of skilled providers in urban areas, inconsistent regulation, and limited public-sector absorption of graduates (10, 34).

Community health workers and female health workers are among Somalia's most important resilience assets. Evidence from polio surveillance and COVID-19 response shows that community-based workers can extend surveillance, household screening, referral, and risk communication (41, 42). The new Community Health Strategy is therefore a major policy opportunity (14). Its success will depend on whether the cadre is paid, trained, supervised, supplied, protected, and integrated with PHC facilities and DHIS2 rather than treated as a low-cost substitute for facility readiness (14, 41, 42).

### 3.5 Service delivery and primary health care

Service delivery in Somalia is characterized by a mixed public, private, humanitarian, and NGO landscape (12, 23, 36). The HSSP III and EPHS provide a policy basis for PHC strengthening, but access remains unequal across urban, rural, nomadic, displaced, and conflict-affected populations (10, 13, 23). Private providers are particularly important in urban areas, but weak regulation and incomplete reporting limit quality assurance, referral coordination, outbreak visibility, rational medicine use, and national understanding of service utilization (36).

Private-sector stewardship should therefore be treated as a practical resilience function. Priority mechanisms include licensing and periodic inspection of private facilities, pharmacies, and laboratories; mandatory notification of epidemic-prone diseases; minimum clinical quality and patient-safety standards; standardized referral protocols with public and humanitarian providers; pharmaceutical regulation to reduce unsafe or substandard medicines; laboratory reporting for priority pathogens; and phased integration of private-sector indicators into DHIS2 or a compatible reporting architecture. The aim is not to replace private providers, but to bring them into a common accountability, quality, referral, and surveillance framework (12, 34, 36).

Resilience depends on whether essential services can continue during shocks. In Somalia, immunization, maternal and newborn care, nutrition treatment, outbreak management, tuberculosis services, malaria services, and emergency care are repeatedly disrupted by insecurity, floods, drought, supply shortages, and funding gaps (23, 38, 40, 43). Strengthening PHC is therefore not only a development priority; it is an emergency-preparedness intervention. The broader resilience and high-quality health-system literature supports this position: countries and fragile settings require people-centered, equitable, trusted, and continuously learning primary care platforms to maintain essential services under stress (15–17, 20).

Equity is central to resilience because shocks do not affect all populations equally. IDPs are least protected because they often live in overcrowded settlements with weak WASH, insecure tenure, low income, and limited continuity of care (23, 40, 43). Nomadic and rural populations face distance, transport, insecurity, and weak outreach systems (10, 12). Women and children face gendered access barriers, maternal and newborn risks, and dependence on household decision-making and transport (23, 37). People with disabilities may be excluded from routine outreach, facility access, and emergency communication. In insecure districts, both patients and health workers face mobility constraints, while facility reporting may be incomplete. A resilience dashboard should therefore disaggregate service continuity and outbreak outcomes by district, sex, age, displacement status, disability, rural or nomadic context, and where feasible socioeconomic vulnerability (6, 7, 19).

### 3.6 Health information and surveillance

Health information is one of the clearest areas of progress. A WHO assessment reported that DHIS2 monthly reporting completeness improved from 62% in 2016 to 91% in 2022 (32). This is a substantial resilience-enabling gain because outbreak detection, resource allocation, service continuity monitoring, and accountability depend on routine data (31, 32).

However, routine data limitations remain serious. Private facilities, inaccessible areas, nomadic populations, internally displaced persons, and mortality events are still incompletely captured (31, 32). Chronic diseases, service quality, medicine stockouts, referral completion, disability, and equity indicators are also weakly represented. A resilient HMIS must move beyond aggregate service counts toward timely, usable, disaggregated data that can guide district action (31, 32).

### 3.7 Medical products, supply chains, and infrastructure

Supply-chain reliability is a major bottleneck for resilience (10, 12, 40). During conflict, floods, and drought, transport disruption, insecurity, fragmented procurement, limited warehousing, and dependence on partner supply systems can interrupt vaccines, essential medicines, diagnostics, nutrition commodities, oxygen, and emergency supplies (23, 40, 43). Emergency health support has included oxygen and emergency-response investments, but these need to be institutionalized through national procurement, maintenance, stock monitoring, and contingency planning (40).

Supply-chain resilience should be measured through practical indicators: stockout rates for tracer medicines, cold-chain functionality, time to replenish emergency supplies, availability of oral rehydration salts and intravenous fluids during cholera seasons, oxygen availability in referral facilities, and availability of maternal and newborn commodities. Without these operational indicators, policy commitments remain difficult to verify (6, 9, 10).

Table 2 consolidates the evidence of adaptive capacity, remaining vulnerability, and priority action for each health-system function.

**Table 2:** Resilience capacities and remaining gaps by health-system function.

Function	Evidence of adaptive capacity	Remaining vulnerability	Priority action
Governance	HSSP III, EPHS direction, GFF investment case, and Community Health Strategy provide reform frameworks (10, 12, 14).	Fragmented implementation across federal, state, private, NGO, and humanitarian actors.	Use one plan, one monitoring framework, and one reporting architecture with state-level accountability.
Financing	Donor and humanitarian resources have repeatedly enabled emergency response (10, 12).	Domestic public health financing is low and unpredictable; out-of-pocket spending remains a barrier (47).	Increase predictable domestic financing and align partner funds with national PHC priorities.
Workforce	Community health workers, female health workers, and surveillance volunteers have shown operational value (41, 42).	Workforce density, distribution, regulation, supervision, and retention remain weak (10, 34).	Professionalize cadres, pay and supervise them, and strengthen HRH registries and licensing.
Service delivery	EPHS and PHC reforms define a service-delivery direction (10, 13).	Rural, nomadic, displaced, and conflict-affected communities remain underserved (10, 12, 23).	Invest in PHC readiness, referral systems, outreach, and essential service continuity during shocks.
Information systems	DHIS2 reporting completeness has improved substantially (31, 32).	Private-sector, equity, mortality, NCD, and quality data remain incomplete (31, 32).	Integrate private and humanitarian providers and use district data-review meetings.
Supply chains	Emergency partners maintain critical commodities during outbreaks and crises (10, 12, 40).	Fragmented procurement, stockouts, weak maintenance, and transport disruption persist (23, 40, 43).	Establish tracer commodity monitoring, pre-positioning, and contingency stock systems.
Community engagement	Local workers and community volunteers have supported surveillance, risk communication, and uptake (41, 42).	Mistrust, misinformation, gender barriers, and displacement complicate reach (23, 40, 43).	Institutionalize community engagement through FHWs, religious leaders, youth, and local committees.

EPHS, Essential Package of Health Services; FHW, female health worker; GFF, Global Financing Facility; HRH, human resources for health; NCD, noncommunicable disease; NGO, non-governmental organization; PHC, primary health care.

## 4 Actionable recommendations

The policy task is to convert repeated emergency adaptation into institutional resilience. The following recommendations are policy inferences derived from three evidence streams: Somalia-specific observations of operational capacity and persistent gaps; national strategies and institutional commitments; and wider evidence on resilience in fragile and conflict-affected settings. They should not be interpreted as causal findings that any single reform has already reduced mortality or guaranteed service continuity in Somalia. Each recommendation therefore requires local costing, piloting where appropriate, implementation monitoring, and evaluation before large-scale expansion. Priority is given to actions that can protect essential services, use existing systems, and create the institutional conditions for later reforms.

### 4.1 Establish one national resilience framework with decentralized accountability

The Federal Ministry of Health should lead a single national resilience framework aligned with HSSP III, EPHS, the Community Health Strategy, IDSR/EWARN, and national health-financing reforms (10, 12–14). The framework should define a small set of national service-continuity and preparedness standards while allowing Federal Member States and districts to adapt contingency plans to local risks. Annual national and state scorecards should be paired with quarterly district reviews covering service disruption, workforce availability, stockouts, referrals, outbreak response, and corrective actions. This approach would reduce the current tendency for resilience activities to be distributed across disconnected emergency plans and partner projects.

### 4.2 Shift from reactive appeals to predictable and aligned financing

Somalia should define a medium-term domestic health-financing pathway that first protects primary health care, surveillance, laboratory services, community health, essential medicines, referral capacity, and emergency preparedness. Monitoring should distinguish approved allocation, release, execution, and actual expenditure because an appropriation does not guarantee operational capacity. Each proposed scale-up should identify its recurrent and capital costs, financing source, procurement and workforce implications, and transition arrangement if donor support ends. Donors and humanitarian partners should map funding against national priorities, use common reporting arrangements, and move suitable functions from short project cycles to multi-year support (12, 23, 45, 46, 48–51). Flexible contingency financing should be available before predictable seasonal risks, including cholera and drought-related nutrition emergencies, rather than mobilized only after service disruption and mortality increase. Unfunded mandates—particularly new cadres, reporting requirements, or infrastructure obligations without recurrent financing—should be avoided.

### 4.3 Protect essential services through primary health-care readiness

Each district should maintain a costed continuity plan for immunization, maternal and newborn care, nutrition, tuberculosis, malaria, outbreak management, and emergency referral. Minimum readiness standards should include defined staffing, essential diagnostics, tracer medicines, cold-chain functionality, infection prevention and control, water and power continuity, referral communication, and transport arrangements. Monthly trend monitoring should trigger outreach, redistribution of commodities, temporary redeployment of staff, or referral support when service volumes fall below locally agreed baselines during a shock (10, 13, 15–17, 20, 23). Strengthening primary health-care readiness is therefore both a development intervention and an emergency-preparedness measure.

### 4.4 Professionalize community health platforms

Female health workers, community health workers, and surveillance volunteers should be treated as an integrated component of the health system rather than as an inexpensive substitute for functional facilities. The Community Health Strategy should be operationalized through predictable remuneration, standardized training and certification, supportive supervision, essential supplies, protection, referral feedback, and inclusion of community indicators within DHIS2-compatible reporting (14, 41, 42). District reviews should track household contacts, referrals issued and completed, supervision coverage, and reasons for failed referrals. These measures would preserve the reach demonstrated during polio and COVID-19 responses while reducing dependence on temporary campaign structures.

### 4.5 Integrate private providers into stewardship, referral, and reporting

Private facilities, pharmacies, and laboratories should be progressively incorporated into a shared regulatory and accountability architecture. Priority steps include licensing and periodic inspection, minimum quality and patient-safety standards, mandatory notification of epidemic-prone diseases, standardized referral protocols, pharmaceutical oversight, priority laboratory reporting, and phased submission of core indicators through DHIS2 or an interoperable platform (12, 34, 36). The objective is not to displace private provision but to make it visible and accountable within national planning, surveillance, quality assurance, and continuity arrangements.

### 4.6 Embed climate-health, WASH, nutrition, and displacement preparedness

Climate-health preparedness should be treated as a routine cross-cutting system function. District contingency plans should combine climate and early-warning information with nutrition surveillance, WASH risk mapping, IDSR/EWARN alerts, pre-positioned cholera and nutrition commodities, mobile and outreach capacity, and referral transport. Plans should explicitly identify internally displaced, nomadic, rural, disabled, and otherwise underserved populations because shocks amplify existing access barriers (6, 7, 19, 23, 38, 40, 43). Preparedness targets should be reviewed before high-risk seasons and after each emergency to ensure that response learning changes routine practice.

### 4.7 Operationalize a resilience dashboard linked to decisions

Somalia needs a practical dashboard that triggers action rather than merely describing performance, but it should be introduced in phases. Phase 1 should use a small set of indicators already available from DHIS2/HMIS, IDSR/EWARN, facility registers, budget records, and stock cards, including essential-service trends, alert-to-investigation time, cholera case fatality, reporting completeness, selected stockouts, and budget release or execution. Phase 2 should add standardized community-health reporting, an updated HRH registry, stronger LMIS coverage, referral-completion measures, and phased private-sector reporting. Phase 3 should develop more demanding equity measures, including reliable disaggregation by displacement, disability, and rural or nomadic context, using revised registers, interoperable systems, and targeted population-based data collection where routine denominators are inadequate. Decision thresholds should be piloted and validated before they are used for accountability or resource reallocation. Table 3 therefore distinguishes what can be measured now from indicators requiring system development; Table 4 assigns actions and accountability to the responsible actors.

**Table 3:** Proposed phased monitoring indicators for a Somalia health-system resilience dashboard.

Domain	Core indicator	Current measurability and implementation phase	Data source and frequency	Responsible body	Decision triggered
Essential service continuity	Monthly trends in immunization, ANC, facility delivery, nutrition treatment, TB services, and OPD visits during shocks	Phase 1: measurable for public and participating partner facilities through DHIS2/HMIS. Phase 2: expand private and humanitarian reporting. Phase 3: strengthen IDP and nomadic denominators.	DHIS2/HMIS and facility registers; monthly	FMoH HMIS unit, FMS health information teams, district health offices	After baseline validation, if service volume falls below the locally agreed range during a shock, activate the continuity plan, outreach, referral support, or commodity redistribution.
Outbreak response	Time from alert to investigation; time from confirmation to response; cholera CFR	Phase 1 where IDSR/EWARN is functional. Investigation-time and CFR thresholds require baseline validation and review of case ascertainment and reporting completeness.	IDSR/EWARN, outbreak line lists; weekly during outbreaks	FMoH surveillance unit, FMS surveillance teams, district rapid response teams	If validated alert-investigation or CFR thresholds are exceeded, review data quality and deploy rapid-response, WASH, laboratory, and case-management support as indicated.
Community reach	FHW/CHW household visits, referrals issued, referrals completed and supervision sessions	Phase 2: partly available in programme registers, but national use requires standardized community definitions, supervision tools, referral feedback, and DHIS2 configuration.	Community registers, supervisor reports, DHIS2 community module; monthly	Community health programme, district health offices, implementing partners	If standardized supervision or referral-completion indicators deteriorate, identify the cause and strengthen supervision, transport linkage, supplies, or facility feedback.
Workforce	Availability of staff by cadre, facility, district and shift; vacancy and absenteeism rates	Phase 2: facility staffing can be assessed, but vacancy, shift coverage, and absenteeism require an updated HRH registry, standard definitions, and routine roster verification.	HRH registry, facility assessment, supervision reports; quarterly	FMoH HRH directorate, FMS HRH units, facility managers	If verified critical vacancies, shift gaps, or absenteeism rise, consider redeployment, surge contracts, retention support, or hard-to-reach incentives within available financing.
Financing	Budget allocation, release, execution and proportion spent on PHC and preparedness	Phase 1 for approved allocation and release. Phase 2 for execution, expenditure by function, recurrent-cost tracking, and harmonized donor and off-budget mapping.	Government budget, expenditure reports, donor mapping; quarterly/annual	FMoH finance/planning units, Ministry of Finance, donors	If approved releases or execution fall below agreed milestones, conduct a joint budget review and prioritize protection of essential services before adding new commitments.
Supply chain	Stockout days for tracer medicines, vaccines, ORS, IV fluids, oxygen, diagnostics and maternal commodities	Phase 1 where facility stock cards are maintained. Phase 2 requires broader LMIS coverage, common tracer lists, and validated stockout-day definitions.	LMIS, facility stock cards, supervision; monthly	National supply-chain unit, FMS logistics teams, facility pharmacists	If validated tracer-stockout days exceed the agreed threshold, initiate redistribution, emergency procurement, or pre-positioning and document resolution time.
Data quality and private-sector reporting	Reporting completeness, timeliness, internal consistency, private-sector reporting and data-use meetings	Phase 1 for public and participating partner facilities. Phase 2 for licensed private providers after reporting standards, agreements, support, and enforcement mechanisms are established.	DHIS2 analytics, data-quality audits, private-facility reports; monthly/quarterly	FMoH HMIS unit, FMS HMIS teams, private-sector focal points	If reporting falls below the agreed target, conduct a data-quality review, mentorship, and phased enforcement after facilities have received standards and reporting support.
Equity	Service coverage and outbreak outcomes by district, sex, age, IDP status, disability and rural/nomadic context	Phase 3 developmental indicator. Reliable disability, displacement, and nomadic disaggregation requires revised registers, privacy safeguards, interoperable identifiers, surveys, and valid population denominators.	HMIS, surveys, partner reports, facility/community registers; quarterly/annual	FMoH planning and HMIS units, FMS and district teams, partners	If validated data show a persistent coverage or outcome gap, target outreach, mobile services, fee protection, referral support, or further assessment of the affected population.

ANC, antenatal care; CFR, case-fatality ratio; CHW, community health worker; DHIS2, District Health Information Software 2; EWARN, Early Warning, Alert and Response Network; FHW, female health worker; FMoH, Federal Ministry of Health; FMS, Federal Member State; HMIS, Health Management Information System; HRH, human resources for health; IDP, internally displaced person; IDSR, Integrated Disease Surveillance and Response; IV, intravenous; LMIS, Logistics Management Information System; OPD, outpatient department; ORS, oral rehydration salts; PHC, primary health care; TB, tuberculosis. Phase 1 uses existing routine sources with limited modification; Phase 2 requires strengthened registries, interoperability, private-sector or community reporting, and additional supervision; Phase 3 requires substantial system development or population-based data. All proposed triggers are illustrative and should be applied only after pilot testing, denominator and data-quality validation, assignment of decision authority, and confirmation that resources are available for the required response.

**Table 4:** Actor-specific policy actions to convert emergency adaptation into institutional resilience.

Actor	Priority action	Practical accountability measure
Federal Ministry of Health	Lead a phased and costed national resilience framework aligned with HSSP III, EPHS, community health, IDSR/EWARN, and financing reforms	Annual resilience review using a limited Phase 1 dashboard, costed implementation plan, and national/state scorecards
Ministry of Finance and Parliament	Protect priority health functions in the medium-term expenditure framework; publish allocation, release, execution, special-project, and recurrent-cost information	Quarterly budget execution review and annual disclosure of recurrent versus project financing for essential services and preparedness
Federal Member States and districts	Translate national priorities into district contingency plans, PHC readiness targets, referral routes and data-use meetings	Quarterly district reviews documenting service continuity, stockouts, referral completion and outbreak response
Donors and humanitarian partners	Align funding with one national plan, disclose programme gaps and recurrent costs, reduce parallel reporting, and provide transition plans for functions expected to be absorbed by government	Donor mapping showing duration, recurrent-cost coverage, alignment with PHC and preparedness priorities, and an agreed transition or closure plan
Private providers	Participate in licensing, clinical standards, mandatory disease reporting, referral protocols, pharmaceutical regulation and DHIS2-compatible reporting	Proportion of registered private facilities submitting priority reports and participating in referral/surveillance systems
District health authorities	Maintain PHC readiness, monitor high-risk populations, supervise FHW/CHW platforms and coordinate local WASH/nutrition/outbreak response	Monthly action tracker for underserved districts, IDP settlements, nomadic outreach and stockout resolution
Community platforms	Use FHWs/CHWs, religious leaders, youth and local committees for risk communication, household referral and feedback	Referral completion, supervision coverage and community feedback reviewed in district meetings

CHW, community health worker; DHIS2, District Health Information Software 2; EWARN, Early Warning, Alert and Response Network; FHW, female health worker; HMIS, Health Management Information System; HRH, human resources for health; IDP, internally displaced person; IDSR, Integrated Disease Surveillance and Response; PHC, primary health care; WASH, water, sanitation and hygiene.

### 4.8 Sequence implementation under binding fiscal constraints

The seven priorities are not equally affordable or immediately implementable and should not be launched simultaneously. During Phase 1 (0–24 months), the immediate objective should be to prevent avoidable service collapse: protect existing PHC and outbreak functions, validate 10–15 core resilience indicators, maintain district continuity plans, monitor critical commodities, preserve functioning community-health activities, and review budget release and execution. Phase 2 (2–5 years) should strengthen private-sector reporting, HRH and logistics registries, community information modules, referral communication, supervision, and selected facility readiness inputs such as water, power, cold chain, and infection prevention. Phase 3 (five years or longer) should pursue reforms with high recurrent or infrastructure costs, including comprehensive salaried and insured community cadres, broader pooling and domestic financing, full digital interoperability, advanced equity disaggregation, and major facility and supply-chain upgrades. Progression between phases should depend on demonstrated implementation capacity and identified recurrent financing rather than calendar time alone.

Federal and state implementation plans should specify unit costs, recurrent vs. capital requirements, financing sources, responsible institutions, procurement and workforce dependencies, and measurable milestones. Low-cost governance and reporting reforms may improve coordination, but they cannot substitute for medicines, personnel, transport, WASH, electricity, and referral capacity. Conversely, expanding service inputs without governance, data use, and accountability may reproduce fragmented projects. The proposed sequence is therefore a prioritization framework rather than a costing study; formal programme costing and cost-effectiveness research remain necessary before national scale-up.

## 5 Discussion

### 5.1 What has made Somalia's health system adaptive?

Somalia's health system has survived because it has repeatedly mobilized flexible actors outside conventional public-sector structures (12, 23, 33, 36). Community workers, diaspora professionals, local NGOs, private clinics, United Nations agencies, and donors have filled service gaps. This adaptive pluralism has been lifesaving during acute shocks. It explains why polio surveillance could be strengthened through village volunteers, why cholera mortality declined across repeated waves, and why COVID-19 surveillance could reach households despite limited facility capacity (39, 41, 42).

The second adaptive asset is policy learning. The HSSP III, the investment case, DHIS2 expansion, and the Community Health Strategy show that Somalia is not only responding to emergencies; it is also attempting to rebuild systems (10, 12, 14, 32). Recent Somalia-specific policy analyses similarly emphasize that progress toward transformation and universal health coverage depends on stronger federal-state coordination, health financing, workforce retention, digital-health interoperability, and accountable implementation of national plans (34). These reforms matter because resilience requires institutional memory and strategic direction. However, the policy-to-practice gap remains wide.

### 5.2 What remains fragile?

The main fragility is structural dependence on external and emergency-oriented financing (10, 12, 23). When essential services rely on project cycles, each shock can trigger new resources but not necessarily stronger institutions. The second fragility is fragmentation (12, 23, 34, 36). A system with many providers can expand access, but without common standards, referral pathways, data reporting, and accountability, it becomes difficult to guarantee quality and equity. The third fragility is the limited readiness of PHC facilities (10, 13, 23). Community screening, surveillance, and referral lose credibility when facilities lack medicines, diagnostics, staff, or transport links. These risks mirror wider critiques of resilience discourse, which warn that resilience should not excuse chronic underinvestment or normalize the expectation that frontline workers and communities must continually compensate for weak institutions (24).

A fourth fragility is weak measurement of resilience itself. Somalia has improving DHIS2 reporting, but resilience indicators are not yet routinely institutionalized (31, 32). Reporting completeness alone does not show whether services continued during floods, whether cholera supplies were available before outbreaks, whether displaced households accessed care, or whether health facilities had functioning staff, oxygen, and medicines.

### 5.3 Feasibility of the dashboard and policy recommendations

The feasibility problem is substantive rather than merely technical. The official 2025 federal appropriation for the Ministry of Health was US$91.85 million, with US$68.42 million recorded under special projects, while programme reports in 2025 documented severe health-sector funding shortfalls and closure of supported sites (46, 48, 49). These sources use different denominators and cannot be combined into one estimate of the national financing gap, but together they show that recurrent salaries, supervision, commodities, referral transport, infrastructure maintenance, and information-system expansion face binding resource constraints. The recommendations should therefore be interpreted as a staged transition pathway, not as an assumption that adequate funding is already available.

A minimum dashboard is feasible using existing routine systems, but even Phase 1 requires an agreed indicator dictionary, validated baselines and denominators, clear decision authority, staff time for review, data-quality checks, and a documented response when a threshold is crossed. Phase 2 would require investment in DHIS2 configuration, community registers, HRH and logistics registries, private-provider agreements, training, supervision, and interoperability. Phase 3 equity indicators are the least immediately feasible because disability, displacement, and nomadic status are incompletely recorded and routine facility data may not provide valid population denominators. These indicators should initially be piloted in selected districts and supplemented with surveys or targeted assessments; they should not be presented as nationally objective measures until data completeness, privacy safeguards, and denominator quality are demonstrated.

Under scarcity, the first policy test should be whether an action protects essential services and prevents loss of existing capacity. PHC continuity, outbreak detection and case management, tracer commodities, referral functions, and retention of already trained frontline workers should generally precede more elaborate reporting or institutional expansion. Donors should provide transition plans for functions that government is expected to absorb, while government budgeting should distinguish recurrent commitments from temporary project expenditure. This prioritization does not resolve the financing gap, but it reduces the risk that the dashboard and recommendations become unfunded or unimplementable aspirations.

### 5.4 Interpretation and implications for fragile settings

The evidence supports a cautious interpretation. Somalia has demonstrated operational adaptation, but operational adaptation is not equivalent to institutional resilience. Adaptive pluralism—through community actors, private providers, local organizations, diaspora support, United Nations agencies, and donors—has maintained services and enabled innovation during crisis. Yet the same pluralism can perpetuate fragmentation when funding, workforce incentives, reporting tools, supply chains, and standards remain parallel. The relevant policy test is therefore not whether a response succeeds during one emergency, but whether that response leaves behind stronger public stewardship, more reliable primary care, better financing arrangements, improved information, and greater equity before the next shock. The proposed recommendations are consequently hypotheses for policy and implementation rather than demonstrated causal effects; their value should be assessed through phased implementation, routine evaluation, and Somalia-specific cost and outcome studies. This distinction may also be useful in other fragile and conflict-affected settings where repeated humanitarian response can be mistaken for health-system resilience (7, 21–24, 44, 50).

### 5.5 Research gaps

Several research gaps should be prioritized. First, Somalia needs empirical studies on which investments produce the greatest resilience gains per dollar, including comparisons between PHC readiness, community health workers, WASH integration, surveillance, supply chains, and referral systems. Second, research should examine how social networks, gender norms, clan structures, displacement, and trust influence health-seeking during crises. Third, more studies are needed on the indirect mortality and morbidity effects of drought, conflict, flooding, and service disruption. Fourth, Somalia needs operational research linking climate forecasts, WASH risks, nutrition surveillance, and outbreak early warning. Fifth, routine data-quality studies should assess how well DHIS2 captures private providers, internally displaced persons, nomadic communities, mortality, and service quality. These priorities align with the wider research agenda for fragile settings, which calls for context-sensitive health systems research that can explain not only whether interventions work, but how governance, financing, workforce capacity, and community trust shape implementation under crisis conditions.

### 5.6 Limitations

This review has limitations. It is a narrative policy review rather than a systematic review, and the search strategy was targeted rather than exhaustive. Although eligibility categories, analytical decision rules, and source-selection logic were specified, purposive selection may introduce selection bias, and the absence of independent duplicate screening, inter-rater agreement, and formal quality appraisal limits confidence in weaker sources. Some Somalia policy documents and operational reports may not be publicly available or independently verifiable. The mortality estimates discussed in this review use different populations, periods, counterfactual assumptions, and data sources and therefore cannot be compared as a trend. Routine health data remain incomplete in inaccessible areas, private facilities, nomadic populations, displaced settlements, and mortality reporting; service and outbreak figures should be interpreted cautiously. Grey literature from institutions involved in the response may emphasize programme achievements or use funding denominators that differ from national budgets. The recommendations are inferential and were not derived from an economic evaluation, causal impact analysis, or formal cost-effectiveness assessment. Similarly, the proposed dashboard contains illustrative indicators and triggers; thresholds require baseline validation, piloting, data-quality assessment, and confirmation that responsible institutions have the authority and resources to respond. Finally, because no primary data were collected, this review cannot independently measure service continuity, quality of care, community trust, patient-level outcomes, or the causal effects of reforms.

## 6 Conclusion

Somalia has built important resilience-enabling capacities under prolonged stress, including community surveillance, cholera response, COVID-19 outreach, DHIS2 expansion, strategic planning, and the new Community Health Strategy. These capacities remain incomplete unless they are institutionalized through predictable financing, PHC readiness, professionalized community health systems, integrated data, private-sector stewardship, climate-health preparedness, reliable supplies, and routine monitoring. The transition should be phased and fiscally explicit: protect existing essential services and use currently available indicators first; strengthen registries, private-sector reporting, referral systems, and facility readiness next; and expand high-cost workforce, financing, interoperability, and equity reforms only when recurrent resources and implementation capacity are secured. The central policy task is therefore not to label the system as fully resilient, but to convert repeated emergency adaptation into durable, measurable, and affordable institutional resilience.
